# Episodic Memory Performance Modifies the Strength of the Age–Brain Structure Relationship

**DOI:** 10.3390/ijerph19074364

**Published:** 2022-04-05

**Authors:** Lauren L. Richmond, Timothy Brackins, Suparna Rajaram

**Affiliations:** Department of Psychology, Stony Brook University, Stony Brook, NY 11794-2500, USA; timothy.brackins@stonybrook.edu (T.B.); suparna.rajaram@stonybrook.edu (S.R.)

**Keywords:** episodic memory, adult lifespan, structural neuroimaging, hippocampal volume, cortical thickness

## Abstract

The bivariate relationships between brain structure, age, and episodic memory performance are well understood. Advancing age and poorer episodic memory performance are each associated with smaller brain volumes and lower cortical thickness measures, respectively. Advancing age is also known to be associated with poorer episodic memory task scores on average. However, the simultaneous interrelationship between all three factors—brain structure, age, and episodic memory—is not as well understood. We tested the hypothesis that the preservation of episodic memory function would modify the typical trajectory of age-related brain volume loss in regions known to support episodic memory function using linear mixed models in a large adult lifespan sample. We found that the model allowing for age and episodic memory scores to interact predicted the hippocampal volume better than simpler models. Furthermore, we found that a model including a fixed effect for age and episodic memory scores (but without the inclusion of the interaction term) predicted the cortical volumes marginally better than a simpler model in the prefrontal regions and significantly better in the posterior parietal regions. Finally, we observed that a model containing only a fixed effect for age (e.g., without the inclusion of memory scores) predicted the cortical thickness estimates and regional volume in a non-memory control region. Together, our findings provide support for the idea that the preservation of memory function in late life can buffer against typical patterns of age-related brain volume loss in regions known to support episodic memory.

## 1. Introduction

Aging is associated with many physical and cognitive changes, including changes in episodic memory performance (see [1] for an overview). Healthy older adults on average self-report having more problems with their memory than they used to [2,3] and rate their memories as poorer than those of younger adults [4]. Although only a small subset of older adults meet the criteria for a diagnosis of clinical memory impairment, such as mild cognitive impairment or Alzheimer’s disease [5], many older adults nonetheless report experiencing distress over age-related memory problems [6].

However, not all older adults exhibit significant late-life decline in episodic memory performance. For example, research with Super Agers, or adults aged 80+ years who exhibit episodic memory performance on par with adults aged 50–65 [7], demonstrated the potential for the relative preservation of episodic memory function into late life. Together with their superior episodic memory ability compared with their average-for-age peers, Super Agers also possess higher cortical thickness estimates [7] and slower rates of cortical volume loss [8]. Importantly, the extent to which these beneficial neural markers observed in Super Agers may extend to a sample of cognitively normal older adults with better-than-average memory (but who may not qualify for Super Ager status) is not yet clear. Moreover, the extent to which preservation of episodic memory function into late life is associated with benefits for the neurobiological underpinnings of episodic memory (e.g., hippocampus, prefrontal cortex, and posterior parietal cortex; see [9]) is not yet well understood.

In the context of normative healthy cognitive aging, older adults exhibit a wide degree of variability in episodic memory scores, and some older adults exhibit better-than-average memory function into late life [10]. Even in episodic memory domains where older adults are expected to suffer the largest changes in memory performance, some older adults exhibit remarkable stability of function over the adult lifespan [11]. Together, these data suggest that while memory decline in late life is common, it is not a foregone conclusion that all older adults will experience distressing levels of memory loss. A key question is what differentiates those who exhibit preservation of function into late life compared with those who experience normative, non-clinical age-related changes in memory.

### 1.1. Brain Structures Supporting Episodic Memory

Early evidence for the structural correlates of episodic memory come from studies in patients with brain lesions. For example, groundbreaking case studies of patients H.M. (see [12] for a description) and K.C. (see [13] for a description) advanced our early understanding of brain structures important for supporting episodic memory [14,15], and this tradition has continued to inform our understanding of the neural substrates of episodic memory to this day [16,17,18]. At the same time, beginning in the mid-1990s, functional neuroimaging techniques became more widely available and served to expand the understanding of the cortical and subcortical regions supporting episodic memory processes. Importantly, this technique allowed for the investigation of brain activity during memory tasks in healthy, cognitively normal adults. The results of these studies served to both confirm earlier understanding of subcortical brain regions important for episodic memory from lesion case studies as well as shed light on additional cortical regions involved in episodic memory processes [9]. Importantly, functional imaging studies revealed the critical roles of the prefrontal cortex and posterior parietal cortex in the selection and retrieval of information in episodic memory (see [19,20] for reviews).

At the same time, the utility of fMRI data for understanding cognitive functioning for older adults can be somewhat limited. For example, older adults are known to exhibit lower rates of trial-by-trial BOLD variability compared with young adults [21,22,23,24], and the reduction in trial-by-trial variability in the BOLD signal for older individuals may reduce the sensitivity of this measure to different cognitive operations and processes. As such, the conclusions drawn about neurocognitive functioning in older adults can depend quite substantially on the selection of the measures [25].

More recently, there has been interest in how individual differences in structural brain correlates might relate to cognitive functioning from a broad definition. Specifically, studies have shown that better episodic memory scores have been associated with larger regional volume estimates in the hippocampus [26] and prefrontal cortex [27]. In addition, posterior parietal regions have been shown to exhibit high levels of activity during memory tasks [28,29]. Moreover, functional connectivity in the medial temporal lobe (including the hippocampus) and portions of the prefrontal and parietal cortices have shown stronger coupling in well-performing older adults in a variety of memory tasks (reviewed in [30]). Thus, as one of our aims, we sought to extend past work by examining prefrontal and posterior parietal cortical volumes rather than activity, as the volumes in these regions relate to episodic memory performance across the adult lifespan. Finally, lower cortical thickness estimates in healthy older adults are associated with poorer episodic memory functioning [31]. Importantly, regional volumes may be more closely associated with performance in relevant cognitive domains (see e.g., [26,27]), whereas cortical thickness may be taken as a global marker of brain health (see e.g., [32]), as implicated in memory.

### 1.2. Open Questions and the Current Study

To date, each of the bivariate relationships between age, cortical thickness, and gray matter volumes of relevant regions and episodic memory performance are well understood. However, the relationship between all three factors (age, cortical thickness, and gray matter volume with episodic memory performance) is not understood as well. To this end, we modeled age and episodic memory performance together to predict the gray matter volumes in regions known to support episodic memory in an adult lifespan sample. We further tested this relationship in a more global brain measure (i.e., cortical thickness) and, as a control comparison, in a non-memory region (i.e., putamen). We anticipated that models allowing for an interaction of age and episodic memory scores would best predict the gray matter volumes in the relevant brain regions (e.g., hippocampus, prefrontal cortex, and posterior parietal cortex). We further predicted that this pattern would be specific to regions known to support episodic memory function.

## 2. Material and Method

### 2.1. Participants

The current study includes data from a subsample of adults from the larger Nathan Kline Institute-Rockland Sample (NKI-RS) study initiative. The secondary data analysis plan and procedure for data obtained under the NKI-RS sample reported here were reviewed and approved by the Stony Brook University Institutional Review Board. Volunteers for the NKI-RS were recruited from Rockland County, New York, a suburban and rural county 20 miles northwest of New York City [33]. Rockland County has a population of 311,687 per the 2010 Census (U.S. Census Bureau, 2011). The full NKI-RS dataset includes participant ages ranging from 6 to 85 years of age. The participants in the NKI-RS studies completed a wide variety of behavioral measures and self-report inventories, in addition to taking part in MRI neuroimaging studies (for a detailed description of the included measures, see [33]). The NKI-RS is comprised of various sub-studies, under which the tasks and imaging parameters differed depending on the sub-study focus. The NKI-RS data selected for analysis in the current manuscript included participants drawn from two adult sub-studies under the larger NKI-RS initiative (Discovery and Neurofeedback) based on the tasks and imaging parameters of interest in our study.

Of note, the participants included in the current analysis comprised a large swath of the adult lifespan (participants aged 19+ years of age). The current sample (*n* = 313) consisted of 210 female (67%) and 103 male (33%) participants, with a mean age of 52.62 (SD = 18.51, range = 19–85 years). The participants had an average of 15.79 years of education (SD = 2.30, range = 11–24 years; education data missing for 3 participants). The current sample was 79% White, 14% Black, 5% Asian, and 0.6% American Indian or Alaskan Native (1.6% of participants indicated “other race”), with 8.31% of participants reporting Hispanic or Latino ethnicity.

#### Exclusion Criteria for Current Study

In order to test our hypotheses of interest in a sample of cognitively normal adults, data from participants who reported having previously been diagnosed with any of the following were excluded: neurodegenerative disorder, neurological disorder, pervasive developmental disorder, major primary psychiatric disorder, acquired immunodeficiency syndrome, or stroke. In addition, data from participants who reported having previously sustained a serious head injury with loss of consciousness were excluded. After removing data from participants with disorders known to impact neurological markers or cognitive performance, 95.53% of our sample self-reported generally being in good health. Of the remaining participants (*n* = 14) who reported not being in good health, only 1.92% of our sample (*n* = 6) reported that their self-reported poor health had a large impact on their daily life.

### 2.2. MRI Data Acquisition

The structural neuroimaging data of interest to the current study were collected at the Nathan Kline Institute for Psychiatric Research. The participants underwent a T1-weighted three-dimensional MP-RAGE scan collected with a Siemens Trio 3.0 T scanner. The acquisition parameters for high-resolution gray matter imaging differed slightly across sub-studies (Discovery sub-study: TR = 1900 ms, TE = 2.52 ms, flip angle = 9°, FoV = 250 mm; Neurofeedback sub-study: TR = 2600 ms; TE = 3.02 ms, flip angle = 8°, FoV = 256 mm). All T1-weighted images were obtained with 1-mm isotropic voxels. Volumetric analyses in sub-samples of participants with slightly different image acquisition parameters have been previously reported [34], so participants who completed our relevant measures of interest were included for analysis in the current study regardless of sub-study enrollment.

### 2.3. Cortical and Subcortical Segmentation

Automated segmentation of the T1-weighted image for each participant was performed using FreeSurfer [35,36,37]. Each scan was processed on the same computer and operating system (Linux Ubuntu 18.0) using the “recon-all” command with default system settings to obtain subcortical segmentation. Cortical segmentation was conducted using the Desikan–Killiany cortical atlas to create cortical parcellation statistics for each cortical structure [38].

#### Quality Control Procedure

Upon completion of the automated segmentation for all imaging data, each scan was reviewed to ensure accuracy for segmentation using FreeSurfer’s recommended quality control procedures (see http://surfer.nmr.mgh.harvard.edu/fswiki/, or see [39] for a similar approach). In brief, the following errors were manually searched for and identified using Freesurfer’s Freeview: Skull strip errors, segmentation errors, intensity normalization errors, pial surface misplacements, and topological defects. Each file that contained an error and required correction was manually edited using Freesurfer’s Freeview (*n* = 28) per FreeSurfer’s recommended method, rerun, and inspected to ensure that the error no longer existed. All scans that initially failed quality control were successfully reprocessed and passed the quality control procedure on the second round.

### 2.4. Behavioral Data Selection

The selection of behavioral data began with an evaluation of the full battery of assessments available from the NKI-RS. This battery contains a total of 48 behavioral measures and 7 cognitive tasks [33], each of which was carefully evaluated for its potential relevance to the research question at hand (see http://fcon_1000.projects.nitrc.org/indi/enhanced/assessments/master_list.html for the full list of assessments included in the NKI-RS). Upon a thorough review, performance on the Rey Auditory Verbal Learning Task (RAVLT [40]) was selected as our episodic memory measure. This well-documented test of episodic memory involves an examiner reading aloud a list of 15 semantically unrelated words at the rate of 1 per second, after which the participant is asked to recall all words from the list that they can remember. This procedure is carried out a total of five times. The examiner then presents a second list of 15 unrelated words, allowing the participant only 1 attempt at recall of this new list. Immediately following this, the participant is asked to remember as many words as possible from the initial list. After a 20-min delay, the participant is again asked to recall as many words as possible from the initial list. The participant then completes a recognition memory test for items presented on the initial list [40]. For the purposes of the current study, the delayed list recall (e.g., 20-min delay) of the initial study list was selected to serve as our key measure of episodic memory.

### 2.5. Analysis and Statistical Approach

All statistical analyses were conducted in RStudio [41]. Correlation tests were computed in base R, and regressions were computed using the lme4 [42] and lmerTest [43] packages. Data visualizations were created using the ggplot2 [44] package. In all analyses, age was treated as a continuous variable. Degrees of freedom for model comparisons were determined using Satterthwaite approximation.

To replicate the prior literature, we conducted a series of bivariate correlations between (1) age and scores on our key episodic memory measure (RAVLT 20-min delay), (2) RAVLT performance and hippocampal volume estimates, and (3) age and hippocampal volume estimates. We further tested the correlations between age and cortical thickness as well as RAVLT performance and cortical thickness. We predicted that older age would be associated with poorer RAVLT scores, that higher RAVLT scores would be associated with larger hippocampal volume and cortical thickness estimates, and that older age would be associated with smaller hippocampal volume estimates and lower cortical thickness.

To test our novel hypotheses that the preservation of episodic memory function across the adult lifespan would be related to preservation of the neurobiological substrates of episodic memory, including the hippocampus, prefrontal cortex, and posterior parietal cortex, we built linear mixed effect models to predict the volumes in these regions (corrected for intracranial volume to control for the influence of head size [45]), specifying a random effect of sub-study in each model. Our base model (model 1) predicted brain volumes in relevant regions specifying age as a fixed effect (and a random effect of the sub-study), our second model included fixed effects for the age and RAVLT scores (and a random effect of sub-study; model 2), and our third model specified an interaction of the fixed effects of age and RAVLT scores (and a random effect of sub-study; model 3). We predicted that models including age and memory score as predictors would explain more variance in the relevant regional volumes compared to a model containing age alone, indicating that preservation of episodic memory function into late life is associated with the maintenance of brain volumes in relevant brain regions. 

To extend the past work on Super Agers regarding higher cortical thickness estimates for individuals displaying relative preservation of memory function into late life, we built a series of linear mixed models to predict cortical thickness estimates from a fixed effect of age (and random effect of sub-study; model 1), from fixed effects of the age and memory scores and a random effect of the sub-study (model 2), and from the interaction of fixed effects of age and memory scores (and a random effect of sub-study; model 3). If previous patterns identified in Super Agers extended to healthy older adults with relatively better average-for-age memory scores (but which may not rise to the level of Super Ager status), we would expect that models 2 and 3 would provide a better fit than model 1. If instead this pattern does not extend to the general population, model 1 would then be expected to provide the best fit.

We further tested the specificity of these patterns by conducting a similar analysis for the putamen (corrected for intracranial volume). Though the putamen has been ascribed a role in stimulus–response learning (see [46] for an overview), we did not expect to observe similar patterns in this region for performance in the episodic memory task we included. Instead, we predicted that neither models 2 (age and RAVLT score) nor 3 (interaction of age and RAVLT score) would fit better than the base model (model 1) predicted by age alone.

## 3. Results

### 3.1. Bivariate Correlations

**Figure 1:**
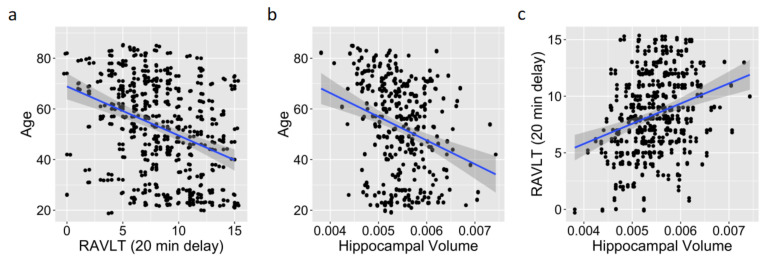
Replication of known bivariate relationships between age, hippocampus, and memory scores, showing replications of known bivariate relationships: a negative relationship between age and memory performance (e.g., RAVLT delayed memory) (**a**), a negative relationship between age and hippocampal volume (**b**), and a positive relationship between memory performance (e.g., RAVLT delayed memory) and hippocampal volume (**c**). Data from the full age range of our sample (19–85 years) are depicted.

As predicted, older age was associated with poorer RAVLT scores (*r* (311) = −0.36, *p* < 0.001, 95% CI [−0.45, −0.26]; Figure 1a) and with smaller hippocampal volume estimates (*r* (311) = −0.28, *p* < 0.001, 95% CI [−0.38, −0.18]; Figure 1b), whereas higher RAVLT scores were associated with larger hippocampal volume estimates corrected for the intracranial volume (*r* (311) = 0.29, *p* < 0.001, 95% CI [0.19, 0.39]; Figure 1c). Higher RAVLT scores were associated with higher estimates of cortical thickness (*r* (311) = 0.22, *p* < 0.001, 95% CI [0.11, 0.32]), and older age was associated with lower cortical thickness estimates (*r* (311) = −0.56, *p* < 0.001, 95% CI [−0.63, −0.47]), which again was in line with the predictions. Importantly, the correlations reported here were consistent with the past literature [26,31].

### 3.2. Hippocampus

**Figure 2:**
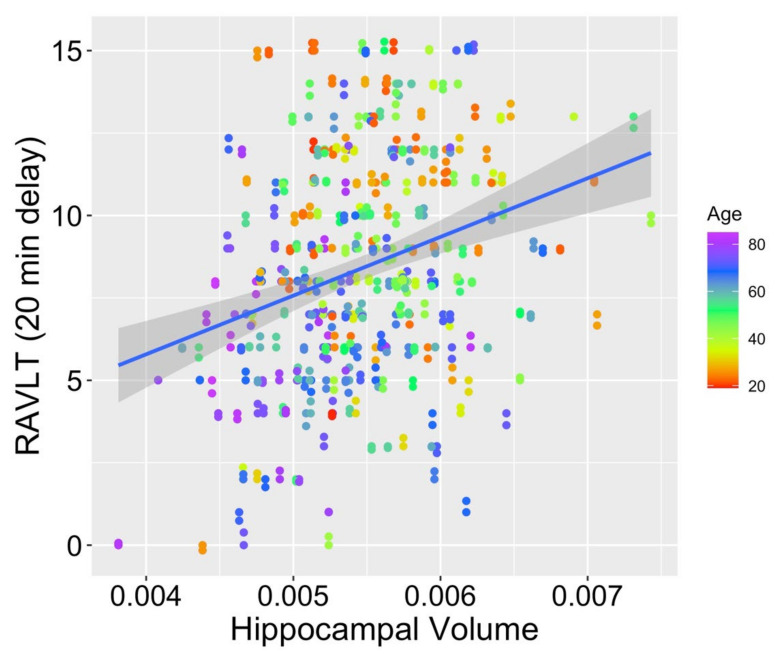
Scatterplot depicting memory score, age, and hippocampal volume. *Note:* scatterplot depicts novel integration of age, memory score, and hippocampal volume within a single model. The full age range of our sample (19–85 years) is depicted in this Figure, with data from participants aged under 20 years appearing in darker red colors and data from participants aged above 80 years appearing in brighter purple.

When comparing the fits for hippocampal volumes (M _hippocampal volume_ = 0.005; SD _hippocampal volume_ < 0.001) from model 1 (age as the only fixed effect), model 2 (age and RAVLT score as fixed effects), and model 3 (interaction of the fixed effects of age and RAVLT score), model 2 was found to fit significantly better than model 1 (χ^2^ (1) = 14.63, *p* < 0.001), as did model 3 compared with model 1 (χ^2^ (2) = 18.82, *p* < 0.001). Model 3 also provided a significantly better fit to the data than model 2 (χ^2^ (1) = 4.19, *p* = 0.041; see Figure 2 for a scatterplot depicting the relationship between RAVLT score, age, and hippocampal volume). Within model 3, both age (*t* (308.6) = −3.28, *p* = 0.001) and the interaction of age and memory scores (*t* (308.8) = 2.07, *p* = 0.039) were significant contributors to the overall model. Memory score alone (*t* (309.0) = −0.60, *p* = 0.551) was not a significant contributor to the model. This pattern supports our prediction that preservation of memory function across the adult lifespan may mitigate typical age-related volume loss in the hippocampus, a region known to support episodic memory.

### 3.3. Prefrontal Cortex

Turning to the prefrontal cortex (M _prefrontal cortex volume_ = 0.070; SD _prefrontal cortex volume_ = 0.007), we observed a somewhat different pattern than was observed in the hippocampus. Model 2 (age and RAVLT score as fixed effects) fit the data only marginally better than model 1 (age as the only fixed effect; χ^2^ (1) = 3.36, *p* = 0.067). Model 3 (interaction of the fixed effects for age and RAVLT score) was also a marginally better fit than model 1 (χ^2^ (2) = 5.35, *p* = 0.069). However, model 3 did not provide a significantly better fit compared with model 2 (χ^2^ (1) = 1.99, *p* = 0.159). Within model 2, age (*t* (308.7) = −12.41, *p* < 0.001) contributed significantly to the model, whereas memory score (*t* (309.2) = −1.82, *p* = 0.070) was only a marginally significant contributor. While this pattern does not suggest that typical age-related volume loss is modified by preserved memory function, it does suggest that participants who are older and who also show poorer memory function will be expected to display relatively lower prefrontal cortex volumes compared with older participants who exhibit relatively better memory, providing partial support for our prediction. We will elaborate on this point further in the Discussion section.

### 3.4. Posterior Parietal Cortex

In our other cortical region of interest, the posterior portion of the parietal lobe (M _posterior parietal cortex volume_ = 0.034; SD _posterior parietal cortex volume_ = 0.003), the pattern of results was similar to the pattern observed in the prefrontal cortex. Model 2 (age and RAVLT score as fixed effects) fit the data significantly better than model 1 (age as the only fixed effect; χ^2^ (1) = 6.32, *p* = 0.012). Model 3 (interaction of fixed effects of age and RAVLT score) was also found to fit significantly better than model 1 (χ^2^ (2) = 6.43, *p* = 0.040). However, model 3 did not provide a significantly better fit compared with model 2 (χ^2^ (1) = 0.11, *p* = 0.741). Within model 2, both age (*t* (262.0) = −6.89, *p* < 0.001) and memory score (*t* (309.6) = 2.48, *p* = 0.014) were significant contributors to the overall model. We again found partial support for our prediction that participants’ memory performance may provide better predictive power over and above models with age alone, suggesting a role for memory performance in refining the understanding of typical age-related volume loss in episodic memory-relevant regions.

### 3.5. Cortical Thickness

A comparison of fits for cortical thickness estimates (M _cortical thickness_ = 2.505; SD _cortical thickness_ = 0.101) from model 1 (age as the only fixed effect), model 2 (age and RAVLT score as fixed effects), and model 3 (interaction of fixed effects for age and RAVLT score) revealed that the fit was not improved for the more complex models (models 2 and 3) over model 1 (model 1 vs. model 2: χ^2^ (1) = 0.19, *p* = 0.663; model 1 vs. model 3: χ^2^ (2) = 0.33, *p* = 0.849). These findings suggest that the relationship between memory preservation and cortical thickness previously observed in Super Agers [7] does not extend to normal variability in memory performance in a population of typical older adults. We return to this finding in the Discussion section.

### 3.6. Control Region: Putamen

In the control region of the putamen (M _putamen volume_ = 0.006; SD _putamen volume_ < 0.001), a very different pattern emerged compared with the patterns observed for the neural substrates of episodic memory. Model 2 (age and RAVLT score as fixed effects) did not provide a better fit to the data over model 1 (age as the only fixed effect; χ^2^ (1) = 3.05, *p* = 0.081), nor did model 3 (interaction of age and RAVLT score; χ^2^ (2) = 3.64, *p* = 0.162). Model 3 also did not provide a significantly better fit compared with model 2 (χ^2^ (1) = 0.587, *p* = 0.444). Together, these data suggest that putamen volume is best predicted by age alone. Furthermore, the patterns reported here, together with those reported for the other cortical and subcortical volumes above, suggests a degree of specificity for the role of memory scores in improving model fits in neural regions known to support episodic memory. We elaborate on this point further below.

## 4. Discussion

In general, our data were consistent with the idea that preserved episodic memory function across the adult lifespan may serve to protect against typical age-related volume loss in brain regions known to support episodic memory. This pattern was observed strongly in the hippocampus, where the age and memory score interacted to predict the hippocampal volume. We observed a similar but more subtle pattern in cortical regions known to support episodic memory performance (prefrontal cortex and posterior portions of the parietal cortex), where both age and episodic memory score predicted the volumes in these regions and did so better than age alone. Importantly, this effect was not observed in a control region—the putamen—where the model with age alone best predicted the structural volume.

Interestingly, we observed that while cortical thickness was separately associated with age (negatively) and memory score (positively), mixed effect models including memory score did not predict cortical thickness estimates better than age alone. This pattern is inconsistent with the findings from Super Agers [7] and therefore suggests that highly superior memory performance may be necessary in order to observe significant associations with this global marker of brain maintenance. It is also possible that the patterns observed in Super Agers may not be present in earlier stages of the adult lifespan, as Super Agers are adults aged 80+ years, and here we included a sample from across the adult lifespan (19+ years of age). In other words, the degree to which the preservation of episodic memory may be associated with benefits to cortical thickness may not be evident until later in life. Future work should address this possibility in a sample constrained to older adults.

The current analysis was conducted on a large-scale dataset collected by the Nathan Kline Institute. As interest in understanding individual differences among participants as they relate to the connection between cognition and neurobiology has increased (see [47] for a discussion), so too has the need for large-scale datasets to address these sorts of questions. This increasing interest in open science practices [48] and data sharing [49] presents opportunities for researchers to address research questions centered on individual differences by using secondary data analysis approaches. Although secondary data analysis may currently be less commonly used in cognitively oriented research compared with other subfields in psychology and neuroscience, this approach is expected to become more popular in the cognitive sciences as open science practices and big data approaches become more common.

These data dovetail with a wealth of literature on structural brain correlates in aging populations, typically depicting an average decline in both cognitive functions and structural brain volumes in older adults (see [50]). Our findings speak to the specificity of the brain–behavior relationship between episodic memory performance and the neural correlates of episodic memory and therefore offer an interesting new perspective suggesting that for older adults who exhibit maintenance of episodic memory function, the negative impact of advancing age on the brain’s structure may be reduced. Our data speak to the potential for lifestyle factors, such as engaging in cognitively stimulating activities, to modify typical age-related trajectories in regional brain volume loss [51,52]. Importantly, although experience-dependent hippocampal plasticity is heightened in childhood [53], hippocampal plasticity is maintained throughout the lifespan. The maintenance of malleability in this region raises the possibility that the structure of the hippocampus will be particularly sensitive to the impacts of both positive (e.g., cognitive stimulation) and negative (e.g., stress) experiences [53] across the lifespan. Thus, having more positively oriented stimulation, avoiding negatively oriented stimulation, or striking an appropriate balance between these two possibilities, throughout development may be associated with the preservation of hippocampal structure later in life. Longitudinal studies may shed further light on the accumulation of lifetime experience as it relates to hippocampal volume maintenance in old age.

The fact that our predicted pattern was supported most strongly in the hippocampus, a region known to be central for supporting episodic memory, was not surprising. What was somewhat surprising at first glance was the relatively more subtle support for this pattern in other cortical regions of interest. However, a wide variety of cognitive functions has been ascribed to these cortical regions, which may perhaps differ from our key cognitive variable of episodic memory and may on its own have some impact on the brain’s structure. For example, the prefrontal cortex has been implicated in cognitive control [54,55], working memory [56,57], and attention [58], cognitive domains that are related to but are also distinct from recall of information in episodic memory per se. Similarly, the posterior parietal cortex has been ascribed roles in spatial attention shifts [59,60] and in route planning [61], both of which differ substantially from our cognitive measure of interest. Additional work to account for other cognitive variables of interest may be warranted to specify other potential sources that could impact the trajectories of volumetric preservation across the adult lifespan.

### Limitations

In the current study, we undertook a correlational design, and therefore, by definition, this design did not include a control group. One of our main variables of interest, namely age, was a continuous variable, for which we also measured memory ability and volumes in relevant brain regions in a continuous fashion. To include a different measure of control in the context of this design, we tested a control brain region instead, namely the putamen, to provide a comparison. Together, these analyses of different sets of brain regions provide insight into the specificity for the observed relationships between age and memory performance with the neural correlates of episodic memory; that is, our key indices do not simply predict overall larger brain volumes. Instead, the predictive power for our variables of interest is only observed in regions that are known to subserve episodic memory function.

One open question that our data cannot speak to is the directionality of this relationship; that is, is it the case that maintaining episodic memory ability later into life confers protection for the relevant neurobiological structures, or does the maintenance of volumes in the relevant brain structures later in life support episodic memory function? Our findings motivate future work involving longitudinal data that may serve to shed light on the directionality of this relationship.

Another limitation of the current work to consider is that the statistical models reported here did not include indicators for overall cognitive health. Importantly, all participants included in the current sample were free from issues known to impact cognitive and neurological functioning, and the participants in this sample were in good physical health overall. Therefore, all participants in this sample would be expected to be in good cognitive health and within the normal range for overall cognitive functioning. Moreover, our findings were specific to regions known to support episodic memory function and did not emerge in our control region (the putamen), suggesting that the patterns reported here may be tied to specific cognitive processes supported by those regions (i.e., episodic memory) and may not emerge when using a global marker of cognition as a predictor. Future work may include a more mixed sample of adult participants, including those with mild cognitive impairment or early Alzheimer’s disease, to better capture the structural brain markers associated with overall cognitive health in a sample that is expected to exhibit more variability in a cognitive health variable and to further investigate the specificity of the patterns observed for our episodic memory performance variable and structural integrity in regions known to support episodic memory.

## 5. Conclusions

Our data suggest that the preservation of episodic memory function into old age is associated with benefits to regional brain structures known to support episodic memory. Memory scores may modify the typical age-related trajectory of brain volume loss, such as was observed in the hippocampus, or may add explanatory power to the statistical model over and above age alone, as was observed in the additional cortical regions of interest. This effect was specific to regions associated with episodic memory, as only age explained the volumes in our non-memory control region (i.e., putamen). Moreover, variability in episodic memory performance exhibited in a typically functioning adult sample aged 19 years and older did not explain variance in one global brain marker: Cortical thickness. Together, these results suggest that the preservation of episodic memory into late life is associated with benefits for brain maintenance in regions known to support episodic memory functioning.

## Data Availability

Restrictions apply to the availability of these data. Data were obtained from the Nathan Kline Institute-Rockland Sample study team and are available upon request from http://fcon_1000.projects.nitrc.org/indi/enhanced/access.html. Data analysis scripts and outputs are available upon request from the corresponding author.
